# Supplementary figures and images for: Development and internal validation of prediction models for future hospital care utilization by patients with multimorbidity using electronic health record data
Source: PLoS One. 2022 Mar 17;17(3):e0260829. doi: 10.1371/journal.pone.0260829 (PMC8929569; doi:10.1371/journal.pone.0260829)

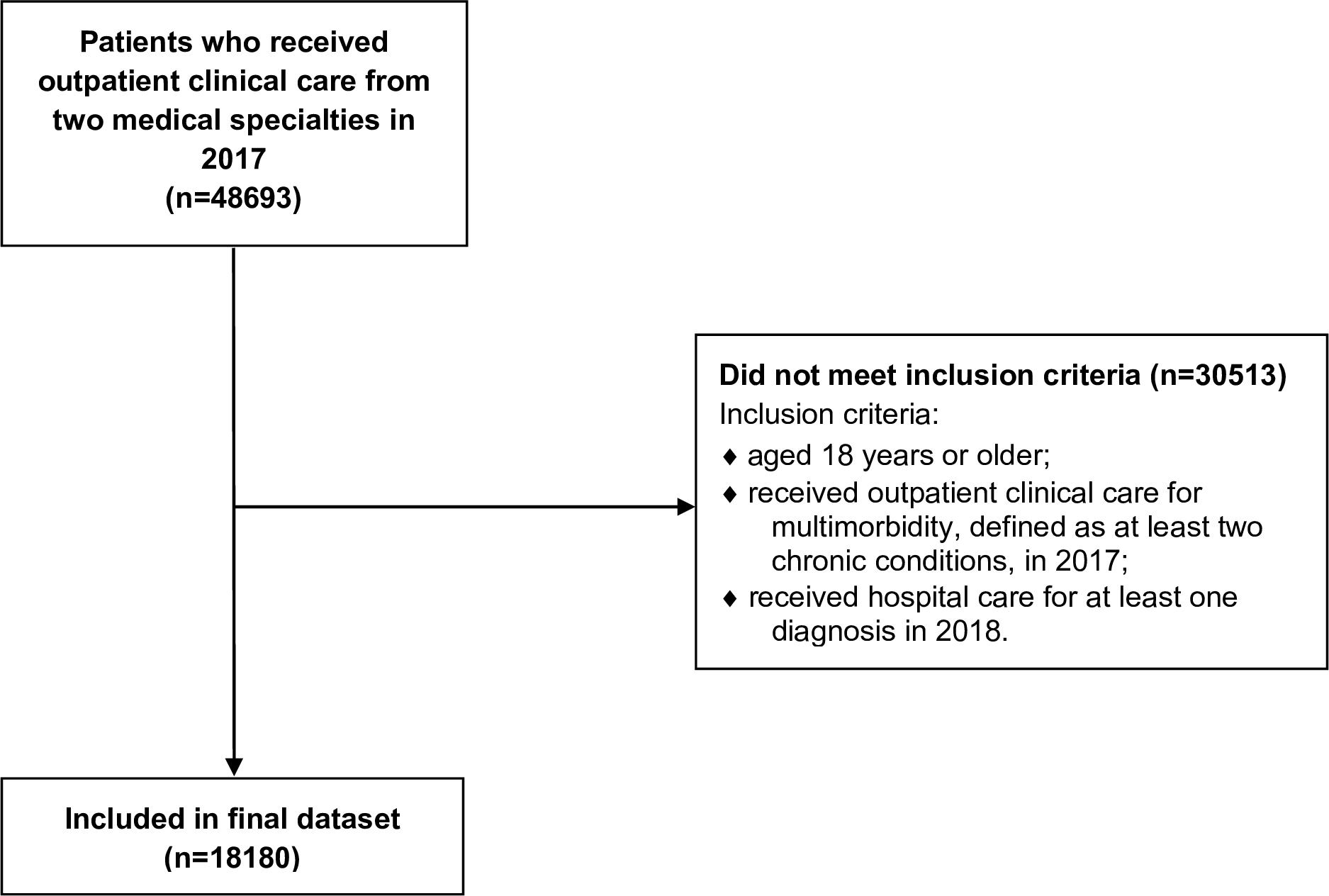

Supplement: S1 Fig — (TIF) [file pone.0260829.s001.tif]

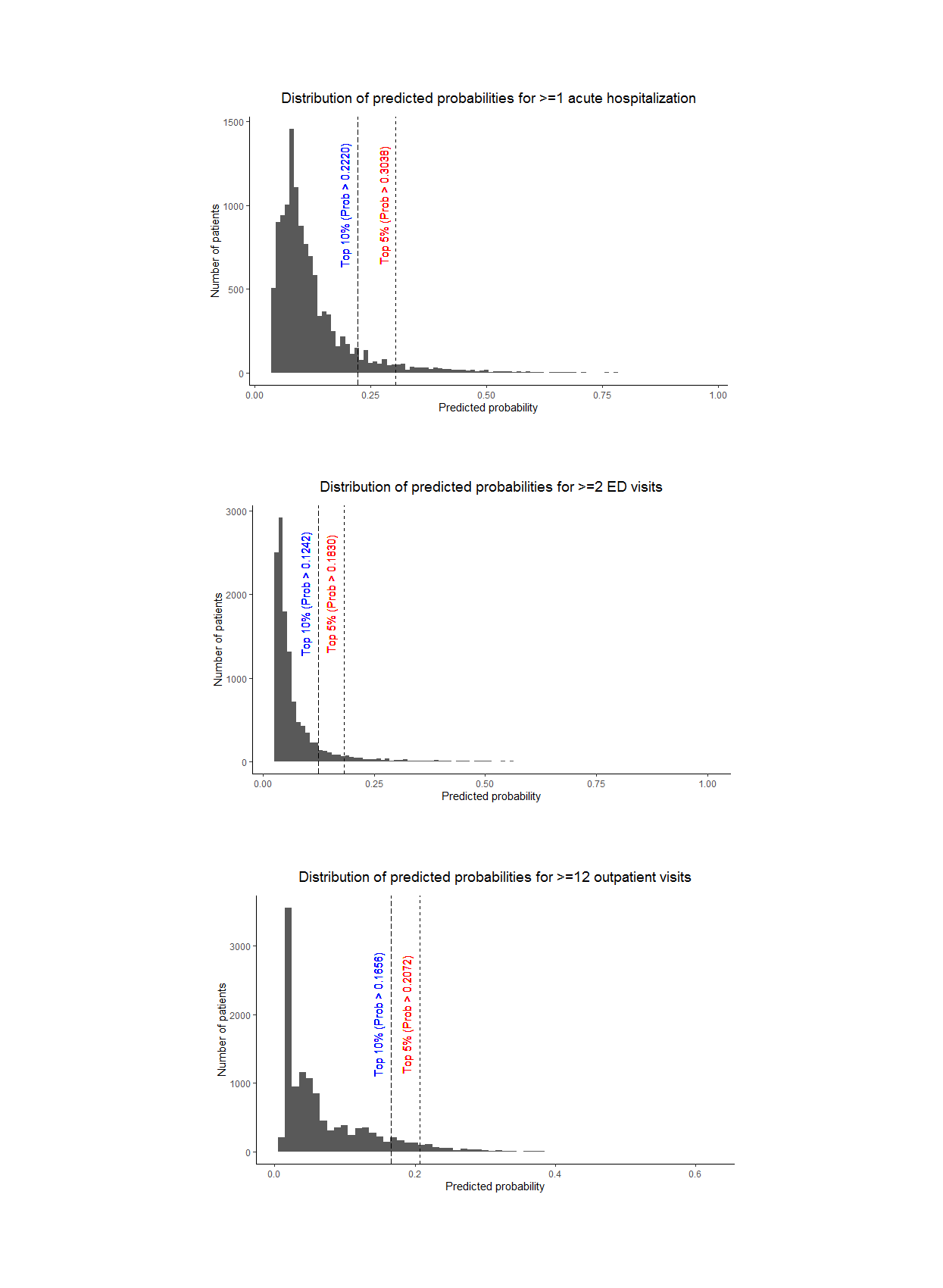

Supplement: S2 Fig — (TIF) [file pone.0260829.s002.tif]
