# Supplementary material for: Development and internal validation of prediction models for future hospital care utilization by patients with multimorbidity using electronic health record data
Source: PLoS One. 2022 Mar 17;17(3):e0260829. doi: 10.1371/journal.pone.0260829 (PMC8929569; doi:10.1371/journal.pone.0260829)
Supplement: S1 Table — Total dataset (n = 18180) was split randomly three times, weighted for every outcome. (PDF) [file pone.0260829.s003.pdf]

Supplementary table 1. Comparison of Development and Validation Data. Total dataset (n=18180) was split randomly three times, weighted for each outcome

Variables	≥ 1 hospitalization		≥2 ED visits		≥ 12 outpatient visits	
	Development	Validation	Development	Validation	Development	Validation
	n=12120	n= 6060	n=12121	n= 6059	n=12120	n= 6060
**Outcome (2018), n (%)**						
≥ 1 hospitalization	1505 (12.4)	752 (12.4)				
≥ 2 ED visits			839 (6.9)	419 (6.9)		
≥ 12 outpatient visits					862 (7.1)	431 (7.1)
**General characteristics (2017)**						
Age group, n(%)						
18-54 years	2795 (23.1)	1337 (22.1)	2686 (22.2)	1446 (23.9)	2818 (23.3)	1314 (21.7)
55-64 years	2407 (19.9)	1178 (19.4)	2328 (19.2)	1257 (20.7)	2367 (19.5)	1218 (20.1)
65-74 years	3569 (29.4)	1800 (29.7)	3707 (30.6)	1662 (27.4)	3557 (29.3)	1812 (29.9)
≥75 years	3349 (27.6)	1745 (28.8)	3400 (28.1)	1694 (28.0)	3378 (27.9)	1716 (28.3)
Sex, female, n (%)	6889 (56.8)	3400 (56.1)	6816 (56.2)	3473 (57.3)	6877 (56.7)	3412 (56.3)
Socioeconomic status, n (%)						
Low	4815 (39.7)	2428 (40.1)	4800 (39.6)	2443 (40.3)	4891 (40.4)	2352 (38.8)
Middle	4839 (39.9)	2405 (39.7)	4891 (40.4)	2353 (38.8)	4827 (39.8)	2417 (39.9)
High	2466 (20.4)	1227 (20.3)	2430 (20.0)	1263 (20.8)	2402 (19.8)	1291 (21.3)
**Disease characteristics (2017)**						
Chronic/oncologic diagnoses, n (%)						
2 chronic/oncologic diagnoses, n(%)	7487 (61.8)	3716 (61.3)	7394 (61.0)	3809 (62.9)	7506 (61.9)	3697 (61.0)
3 chronic/oncologic diagnoses, n(%)	2964 (24.5)	1469 (24.2)	3002 (24.8)	1431 (23.6)	2925 (24.1)	1508 (24.9)
4 chronic/oncologic diagnoses, n(%)	1063 (8.8)	590 (9.7)	1104 (9.1)	549 (9.1)	1104 (9.1)	549 (9.1)
5 chronic/oncologic diagnoses, n(%)	391 (3.2)	184 (3.0)	399 (3.3)	176 (2.9)	383 (3.2)	192 (3.2)
≥6 chronic/oncologic diagnoses, n(%)	215 (1.8)	101 (1.7)	222 (1.8)	94 (1.6)	202 (1.7)	114 (1.9)
Acute diagnoses, median (IQR), diagnoses	0 (0-1)	0 (0-1)	0 (0-1)	0 (0-1)	0 (0-1)	0 (0-1)
**Hospital care characteristics (2017)**						
Medical specialties involved, n (%)						
2 specialties	3752 (31.0)	1807 (29.8)	3668 (30.3)	1891 (31.2)	3767 (31.1)	1792 (29.6)
3 specialties	3882 (32.0)	1935 (31.9)	3861 (31.9)	1956 (32.3)	3829 (31.6)	1988 (32.8)
4 specialties	2396 (19.8)	1266 (20.9)	2448 (20.2)	1214 (20.0)	2436 (20.1)	1226 (20.2)
5 specialties	1209 (10.0)	588 (9.7)	1217 (10.0)	580 (9.6)	1209 (10.0)	588 (9.7)
≥6 specialties	881 (7.3)	464 (7.7)	927 (7.6)	418 (6.9)	879 (7.3)	466 (7.7)
Outpatient visits, n(%)						
2-4 visits	4250 (35.1)	2087 (34.4)	4192 (34.6)	2145 (35.4)	4270 (35.2)	2067 (34.1)
5-7 visits	4127 (34.1)	2049 (33.8)	4074 (33.6)	2102 (34.7)	4116 (34.0)	2060 (34.0)
≥8 visits	3743 (30.9)	1924 (31.8)	3855 (31.8)	1812 (29.9)	3734 (30.8)	1933 (31.9)
Acute hospitalizations, n (%)						
0 acute hospitalizations	9851 (81.3)	4996 (82.4)	9845 (81.2)	5002 (82.6)	9914 (81.8)	4933 (81.4)
1 acute hospitalization	1704 (14.1)	774 (12.8)	1697 (14.0)	782 (12.9)	1624 (13.4)	855 (14.1)
≥2 acute hospitalizations	565 (4.7)	289 (4.8)	579 (4.8)	275 (4.5)	582 (4.8)	272 (4.5)
Inpatient days, n(%)						
0 inpatient days	8906 (73.5)	4496 (74.2)	8854 (73.0)	4548 (75.1)	8976 (74.1)	4426 (73.0)
1-3 inpatient days	1012 (8.4)	492 (8.1)	1017 (8.4)	487 (8.0)	990 (8.2)	514 (8.5)
4-7 inpatient days	1032 (8.5)	499 (8.2)	1038 (8.6)	493 (8.1)	1021 (8.4)	510 (8.4)
8 ≤ inpatient days	1170 (9.7)	573 (9.5)	1212 (10.0)	531 (8.8)	1133 (9.4)	610 (10.1)
Patients with at least 1 ICU admission, n (%)	166 (1.4)	90 (1.5)	181 (1.5)	75 (1.2)	177 (1.5)	79 (1.3)
Emergency department visits, median (IQR), visits	0 (0-1)	0 (0-1)	0 (0-1)	0 (0-1)	0 (0-1)	0 (0-1)
