# Supplementary material for: Development and internal validation of prediction models for future hospital care utilization by patients with multimorbidity using electronic health record data
Source: PLoS One. 2022 Mar 17;17(3):e0260829. doi: 10.1371/journal.pone.0260829 (PMC8929569; doi:10.1371/journal.pone.0260829)
Supplement: S2 Table — (PDF) [file pone.0260829.s004.pdf]

Supplementary table 2. Full Prognostic Model including intercept and model performance measures for derivation and validation set for outcome measure '≥1 hospitalization(s) in 2018'

Intercept and predictors	Beta	SE	P Value
Derivation cohort model estimates			
Intercept	-2.798	0.1016	
Age group			
18-54 years			
55-64 years	0.4266	0.1022	<0.0001
65-74 years	0.5141	0.0943	<0.0001
≥75 years	0.9302	0.0909	<0.0001
Sex, female	-0.2174	0.0578	0.0002
Socioeconomic status			
Low			
Middle	-0.1195	0.0638	0.0610
High	-0.1789	0.0796	0.0245
Chronic/oncologic diagnoses			
2 chronic/oncologic diagnoses			
3 chronic/oncologic diagnoses	0.1099	0.0712	0.1228
4 chronic/oncologic diagnoses	0.2923	0.0977	0.0028
5 chronic/oncologic diagnoses	0.1216	0.1450	0.4017
≥6 chronic/oncologic diagnoses	0.5894	0.1683	0.0005
Number of acute diagnoses	0.0880	0.0404	0.0296
Outpatient visits			
2-4 visits			
5-7 visits	0.0154	0.0782	0.8441
≥8 visits	0.2345	0.0857	0.0062
Acute hospitalizations			
No acute hospitalizations			
1 acute hospitalization	0.2104	0.1152	0.0679
≥2 acute hospitalizations	0.4365	0.1694	0.0100
Inpatient days			
No inpatient days			
1-3 inpatient days	0.0885	0.1164	0.4469
4-7 inpatient days	0.1784	0.1216	0.1423
≥8 inpatient days	0.3845	0.1310	0.0033
Number of emergency department days	0.2088	0.0372	<0.0001
**Model assessment**			
C-statistic (95% CI)	0.702 (0.688-0.716)		
**Model assessment in the validation cohort**			
N	6060		
Number of events	752		
C-statistic (95% CI)	0.692 (0.671-0.713)		
