# Supplementary material for: Development and internal validation of prediction models for future hospital care utilization by patients with multimorbidity using electronic health record data
Source: PLoS One. 2022 Mar 17;17(3):e0260829. doi: 10.1371/journal.pone.0260829 (PMC8929569; doi:10.1371/journal.pone.0260829)
Supplement: S3 Table — (PDF) [file pone.0260829.s005.pdf]

Supplementary table 3. Full Prognostic Model including intercept and model performance measures for derivation and validation set for outcome measure '≥2 ED visits in 2018'

Intercept and predictors	Beta	SE	P Value
Derivation cohort model estimates			
Intercept	-3.6216	0.1157	
Age group			
18-54 years			
55-64 years	0.1010	0.1278	0.4293
65-74 years	0.2536	0.1133	0.0252
≥75 years	0.3501	0.1127	0.0019
Chronic/oncologic diagnoses			
2 chronic/oncologic diagnoses			
3 chronic/oncologic diagnoses	0.0513	0.0927	0.5799
4 chronic/oncologic diagnoses	0.1472	0.1232	0.2324
5 chronic/oncologic diagnoses	0.0831	0.1749	0.6349
≥6 chronic/oncologic diagnoses	0.6399	0.1861	0.0006
Number of acute diagnoses	0.1040	0.0471	0.0271
Outpatient visits			
2-4 visits			
5-7 visits	0.3059	0.1104	0.0056
≥8 visits	0.5441	0.1163	<0.0001
Inpatient days			
No inpatient days			
1-3 inpatient days	-0.0023	0.1355	0.9862
4-7 inpatient days	0.2164	0.1214	0.0748
≥8 inpatient days	0.3156	0.1202	0.0086
Number of emergency department days	0.4020	0.0374	<0.0001
**Model assessment**			
C-statistic (95% CI)	0.717 (0.698-0.736)		
**Model assessment in the validation cohort**			
N	6059		
Number of events	419		
C-statistic (95% CI)	0.672 (0.644-0.701)		
