# Supplementary material for: Development and internal validation of prediction models for future hospital care utilization by patients with multimorbidity using electronic health record data
Source: PLoS One. 2022 Mar 17;17(3):e0260829. doi: 10.1371/journal.pone.0260829 (PMC8929569; doi:10.1371/journal.pone.0260829)
Supplement: S4 Table — (PDF) [file pone.0260829.s006.pdf]

Supplementary table 4. Full Prognostic Model including intercept and model performance measures for derivation and validation set for outcome measure '≥12 outpatient visits in 2018'

Intercept and predictors	Beta	SE	P Value
Derivation cohort model estimates			
Intercept	-4.1799	0.1416	
Age group			
18-54 years			
55-64 years	0.3625	0.1239	0.0034
65-74 years	0.4595	0.1127	<0.0001
≥75 years	0.2938	0.1155	0.0109
Chronic/oncologic diagnoses			
2 chronic/oncologic diagnoses			
3 chronic/oncologic diagnoses	0.2949	0.0944	0.0018
4 chronic/oncologic diagnoses	0.3547	0.1215	0.0035
5 chronic/oncologic diagnoses	0.4481	0.1639	0.0063
≥6 chronic/oncologic diagnoses	0.7764	0.1946	0.0001
Medical specialties involved			
2 specialties			
3 specialties	-0.0762	0.1272	0.5489
4 specialties	0.0270	0.1367	0.8433
5 specialties	0.2905	0.1511	0.0546
≥6 specialties	0.4653	0.1645	0.0047
Outpatient visits			
2-4 visits			
5-7 visits	0.7577	0.1366	<0.0001
≥8 visits	1.5604	0.1401	<0.0001
Number of emergency department days	0.1225	0.0302	<0.0001
**Model assessment**			
C-statistic (95% CI()	0.745 (0.728-0.762)		
**Model assessment in the validation cohort**			
N	6060		
Number of events	431		
C-statistic (95% CI)	0.750 (0.725-0.775)		
